# Application of Perinatal Derivatives in Ovarian Diseases

**DOI:** 10.3389/fbioe.2022.811875

**Published:** 2022-01-24

**Authors:** Anna Lange-Consiglio, Emanuele Capra, Valentina Herrera, Ingrid Lang-Olip, Peter Ponsaerts, Fausto Cremonesi

**Affiliations:** ^1^ Dipartimento di Medicina Veterinaria, Università Degli Studi di Milano, Lodi, Italy; ^2^ Centro Clinico-Veterinario e Zootecnico-Sperimentale di Ateneo, Università Degli Studi di Milano, Lodi, Italy; ^3^ Istituto di Biologia e Biotecnologia Agraria, Consiglio Nazionale Delle Ricerche IBBA CNR, Lodi, Italy; ^4^ Division of Cell Biology, Histology and Embryology, Gottfried Schatz Research Center, Medical University of Graz, Graz, Austria; ^5^ Laboratory of Experimental Hematology, Vaccine and Infectious Disease Institute (Vaxinfectio), University of Antwerp, Antwerp, Belgium

**Keywords:** perinatal derivatives, ovarian diseases, secretome, extracellular vesicles, animal models

## Abstract

Reproductive diseases could lead to infertility and have implications for overall health, most importantly due to psychological, medical and socio-economic consequences for individuals and society. Furthermore, economical losses also occur in animal husbandry. In both human and veterinary medicine, hormonal and surgical treatments, as well as assisted reproductive technologies are used to cure reproductive disorders, however they do not improve fertility. With ovarian disorders being the main reproductive pathology in human and bovine, over the past 2 decades research has approached regenerative medicine in animal model to restore normal function. Ovarian pathologies are characterized by granulosa cell and oocyte apoptosis, follicular atresia, decrease in oocyte quality and embryonic development potential, oxidative stress and mitochondrial abnormalities, ultimately leading to a decrease in fertility. At current, application of mesenchymal stromal cells or derivatives thereof represents a valid strategy for regenerative purposes. Considering their paracrine/autocrine mode of actions that are able to regenerate injured tissues, trophic support, preventing apoptosis and fibrosis, promoting angiogenesis, stimulating the function and differentiation of endogenous stem cells and even reducing the immune response, are all important players in their future therapeutic success. Nevertheless, obtaining mesenchymal stromal cells (MSC) from adult tissues requires invasive procedures and implicates decreased cell proliferation and a reduced differentiation capacity with age. Alternatively, the use of embryonic stem cells as source of cellular therapeutic encountered several ethical concerns, as well as the risk of teratoma formation. Therefore, several studies have recently focussed on perinatal derivatives (PnD) that can be collected non-invasively and, most importantly, display similar characteristics in terms of regenerating-inducing properties, immune-modulating properties and hypo-immunogenicity. This review will provide an overview of the current knowledge and future perspectives of PnD application in the treatment of ovarian hypofunction.

## Introduction

Reproductive disorders are caused by different factors, including social, genetic, endocrine, physiological, psychological and lifestyle habits (e.g., smoking and alcohol consumption), and may lead to infertility (Agarwal et al., 2021; Farsimadan et al., 2021). In human, both men and women are affected, with a male factor occurrence of around 30–40% and a female occurrence of around 50% (Boivin et al., 2007; Gurunath et al., 2011). Reproductive diseases include abnormal hormone production by gonads (ovaries or testes) or endocrine glands (pituitary, thyroid, or adrenals), but can also be caused by genetic or congenital abnormalities, infections, tumours and disorders of unknown cause. In male, the most common reproductive diseases are oligospermia, poor semen quality, low sperm motility, anatomical defects like block in vas deferens, infections leading to inflammation of seminal vesicles, the epididymis or the prostate, genetic abnormalities like Klinefelter, Chlamydia infections, circumcision, erectile dysfunction, genital herpes, genital warts, gonorrhoea and penis disorders (Boivin et al., 2007; Gurunath et al., 2011). In woman, the most common causes of infertility are ovarian hypofunction, irregular ovulation, poor oocyte quality, blocked fallopian tubes due to infection or endometriosis, uterine fibroids, polycystic ovaries, primary and secondary amenorrhea, pelvic inflammatory disease, hostile cervical mucus, sexually transmitted diseases, gynaecologic cancers as well as side effects of cancer chemotherapy (Boivin et al., 2007). Both male and female infertility has implications for overall health. In fact, compared to the healthy population, female infertility is often associated with other disorders such as higher rates of psychiatric disorders, endometrial cancer, polycystic ovary syndrome and patients are more likely to develop cardiovascular disease and metabolic disorders such as diabetes (Hanson et al., 2017). Male infertility on the other hand is reflected by a higher incidence of cancer.

Reproductive diseases are also subject of veterinary medicine. Reproductive performances (for example in dairy cattle) can be complicated by twinning, dystocia, stillbirth, abortion, retained placenta, metritis and ovary hypofunction. These diseases primarily affect the productivity of dairy cows by decreasing reproductive efficiency, shortening the expected productive lifespan and lowering milk production. Poor reproductive performance is a major cause of involuntary culling with negative influence on the subsequent productivity of dairy herd (Ghavi Hossein-Zadeh, 2013).

Despite assisted reproductive technologies have created enormous expectation for infertility treatment, and both hormonal and surgical treatments have been applied to (partially) cure reproductive disorders in human and veterinary medicine, there is an unmet need for awareness that these disorders do have a wider impact on long-term health in human (Hanson et al., 2017) and on the economic management of dairy farms. Therefore, the needs to identify alternative therapies is growing. Over the past 2 decades, researchers have approached the concept of regenerative medicine to restore normal sexual function and preserve fertility, principally focusing on ovarian diseases that are the main pathology in females, both human and animal.

## Ovarian Diseases

**FIGURE 1:**
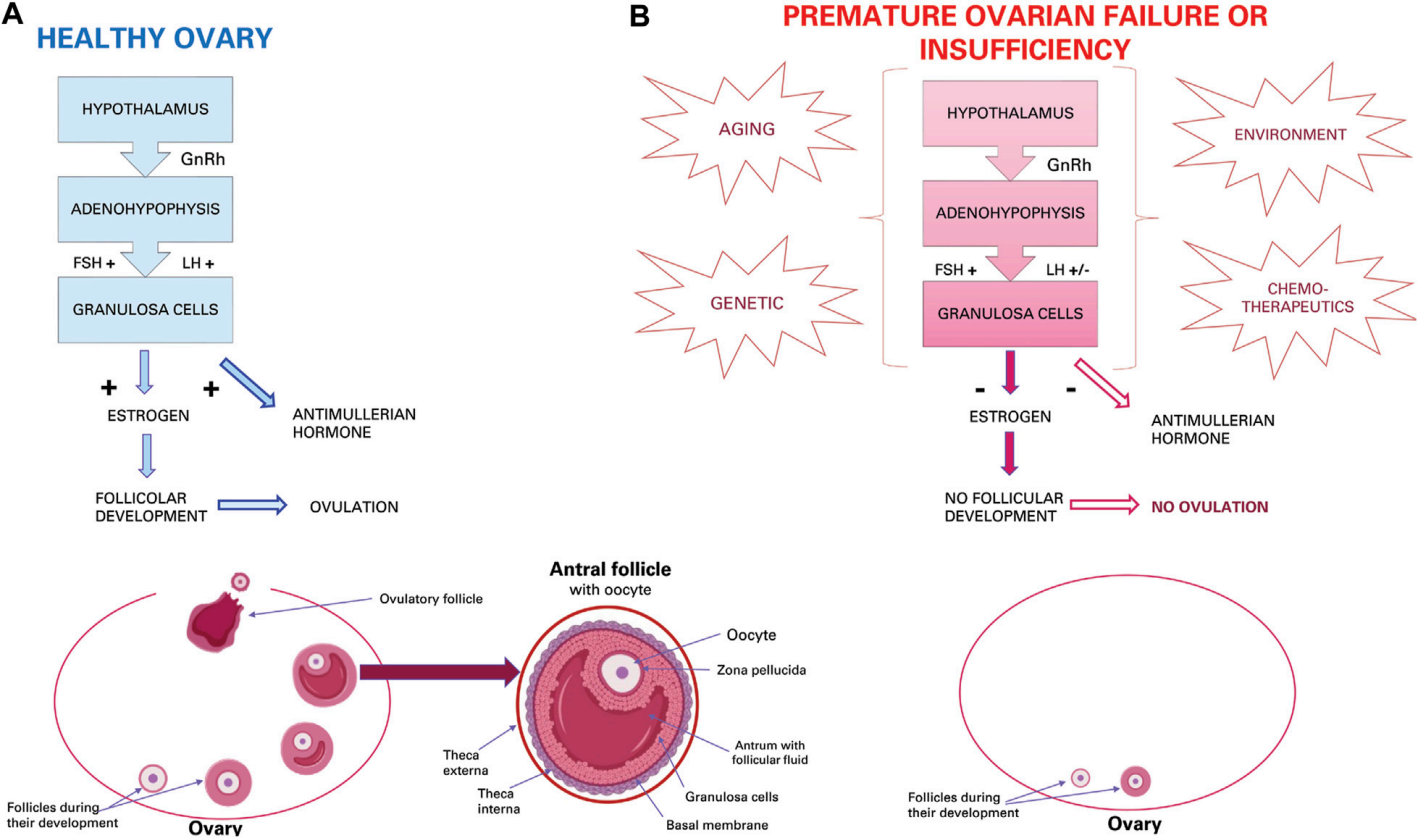
Ovarian activity. **(A)** Physiological hormonal mechanism that induce normal granulosa activity, oestrous cycle, and ovulation. **(B)** Effect of aging, genetic, environmental and chemotherapeutic factors that induce POI or POF.

Functionality of the ovaries through the production of hormones and gametes guarantees the health of the female reproductive system (Kim et al., 2018). In the ovarian follicle, the oocyte is surrounded by granulosa cells and cumulus cells that allow oocytes to survive, grow and mature through molecular and intercellular communications (Thabet et al., 2020). Proliferation and differentiation of granulosa cells is essential for the continuous development of follicles from primary, secondary, tertiary antral follicles up to ovulation (Li et al., 2019). Adequate functioning of the hypothalamus guarantees the hormone-releasing gonadotropins (GnRH) which at the adenohypophysis level will favour the release of follicle-stimulating hormone (FSH) and luteinizing hormone (LH) which are conveyed to the oocyte thanks to the cumulus cells equipped with receptors for these hormones. These two hormones promote the functionality of granulosa cells for the secretion of oestrogen and anti-Mullerian hormone (AMH), and follicular growth until ovulation (Figure 1A). Therefore, proliferation and differentiation of granulosa cells and cumulus cells are basic conditions for the continuous development of ovarian follicles (Zhang H et al., 2018). The AMH, in turn, inhibits the recruitment of primordial follicles from the dormant follicular pool into the emergent pool, thus preserving a state of coordinated ovarian follicular recruitment (Thabet et al., 2020). The AMH, which is expressed in granulosa cells, also participates in the selection of the dominant follicles and plays an important role in follicle growth (Zhang H et al., 2018). In addition, granulosa cells are an important source of oestrogen and progesterone (Li et al., 2019). In the normal developmental period of the ovarian follicular cycle, all phases of follicular atresia are closely related to the activity levels of granulosa cells. In both aging and clinical context, increased apoptosis and decreased proliferation of granulosa cells is the principal mechanism driving follicular atresia and arrest (Ding et al., 2018; Huang et al., 2020). Current knowledge on ovarian dysfunction in the human, murine and bovine reproductive system will be briefly introduced.

### Human Pathology

In situations related to the life of an individual, such as age-related diseases, natural aging, aging induced by therapeutic interventions and diseases, granulosa cells can be prone to persistent senescence (Huang et al., 2020). Physiological ovarian aging is a gradual decrease in both the quantity and quality of oocytes residing within the primordial follicles (Yang et al., 2020) and is linked to ovarian aging characterized by apoptosis of oocytes, follicular atresia, cessation of menstruation and elevated plasma levels of FSH (Ding et al., 2020a; Huang et al., 2020). The condition affects 1% of women worldwide by the age of 40 years (Ding et al., 2020a; Huang et al., 2020).

Menopause in women is an inevitable event due to ovarian aging (Huang et al., 2020), which in mammals implicates the inability to produce new oocytes after birth or during adult life since the number of oocytes is already predetermined as primordial follicles formed before birth. As such, the constant recruitment of primordial follicles and their disappearance over time depletes the ovarian reserve, leading to menopause (Yang et al., 2020). Recently, it has been shown that ovarian dysfunction could also be caused by genetic (e.g., genomic DNA alteration, mitochondrial DNA mutations and reduced telomerase activity) or environmental factors (e.g., oxidative stress, advanced glycation and chemotherapy). Especially for the latter, chemotherapeutic agents either directly affect oocytes or indirectly induce their degeneration by damaging granulosa cells (Hong et al., 2020) (Figure 1B). Furthermore, the process of ovarian dysfunction accelerates with increasing age (Seok et al., 2020) and may lead to various systemic complications (e.g., osteoporosis, heart disease, symptoms of menopause and metabolic syndrome) (Kim et al., 2018).

In addition to the physiological aging of the ovary, also premature ovarian aging may occur and is called “premature ovarian insufficiency” (POI) or “premature ovarian failure” (POF). This pathological condition is characterized by premature depletion of ovarian follicles (Lai et al., 2013). POF affects 1% women under the age of 40 and 0.1% of woman under 30. While about 25% of all forms of POF can be classified as iatrogenic and related to cancer treatment, more than 50% of cases remain idiopathic with unexplained aetiology (Lai et al., 2013). Patients with iatrogenic POF have abnormal ovary development that could be due to genetic disorders, such as fragile X, Turner syndrome, and some autosomal gene mutations (Li et al., 2018). Iatrogenic insults can be represented by cancer treatments, such as chemotherapy or radiotherapy, which can damage ovarian function leading to premature menopause, ovarian dysfunction, and risk of infertility (Li et al., 2018). Idiopathic or iatrogenic POI or POF is clinically characterized by oligomenorrhea or amenorrhea for at least 4 months, high levels of FSH and low levels of 17β-estradiol (E2) (Ling et al., 2019), resulting in oocyte apoptosis, follicular atresia (Ding et al., 2020b), decrease in oocyte quality and embryonic development potential, and, therefore, a decrease in fertility (Liu et al., 2012) (Figure 1B). In addition, the uterus and vaginal mucosa undergo atrophy due to oestrogen deficiency caused by inactive ovaries (Liu et al., 2012). Furthermore, oxidative stress and mitochondrial abnormalities have been shown to be related to follicular atresia and POI (Ding et al., 2020b). Oxidative stress induced by reactive oxygen species (ROS) is usually associated with various age-related diseases. POI is also characterized by decreased antioxidant levels and increased oxidative stress in cumulus cells, oocytes and ovaries, and oxidative stress levels are correlated with worse outcomes (Liu et al., 2012). If POI is not treated, there are many health risks, some of which are typical of menopause such as depression, anxiety, osteoporosis, increased risk of fractures, cardiovascular disease and cognitive decline (Ling et al., 2019). In fact, most women with POF are sterile and suffer from unexpected and early menopause symptoms (Lai et al., 2013). Therefore, in contrast to the inevitable event of physiological ovarian aging, spontaneous premature cessation of ovarian function may need higher clinical and treatment attention in order not to be “permanent” (Li et al., 2018).

### Experimental Models of Ovarian Diseases

Due to the poor feasibility of human studies, it is important to have ideal animal models that mimic the events that occur in human patients with POF or POI.

Usually, the mouse is chosen for its short reproductive life spans, high reproduction index, stable genetic backgrounds, and low costs. In addition, the estrous cycle of female mice is similar to that of humans, although the estrous cycle of mice is shorter than that of humans.

Mouse knockout models have been used to identify genes associated with follicle development, granulosa cell apoptosis, and subsequent damage or depletion of the follicle pool leading to POI or POF (Bouhali et al., 2011).

Since chemotherapy is a common method used to treat various malignancies and could induce ovarian failure and reduce fertility in young female patients (Ling et al., 2019), the treatment of ovarian damage has been studied in animal models, such as mice and rats, in which the disease has been induced by chemotherapy or radiotherapy as reported in Table 1. The most used chemotherapy drugs were cyclophosphamide or busulfan. In some papers, POI in mice was induced by ZP3 peptide, while in other cases, ovariectomized rats, or mice with physiological ovarian aging, or sterilized mice, or rats with naturally aging, or ovariectomized rats were used.

**TABLE 1:** Different sources of perinatal derivatives and their action in the treatment of POI or POF.

Author	Animal	Source	Secretome	Dose	Via	Outcome
Li et al. (2019)	POF mice by ZP3 peptide	hPMSC		1 × 10^6^ hPMSCs	tail vein	hPMSC suppress GCs apoptosis‐induced by ER stress IRE1α signaling pathway contributing to ovarian function recovery
Zhang H et al. (2018)	POF mice by ZP3 protein	hPMSC		1 × 10^6^ hPMSC	tail vein	hPMSC increase the expression of *AMH* and *FSHR*; decrease the level of FSH, LH and AZPAb; increase the level of E2 and AMH; decrease granulosa cell apoptosis; lower number of atretic follicles but more normal follicles
Seok et al. (2020)	Ovariectomized rat model	hPMSC		5 × 10^5^/ml hPMSC	tail vein	hPMSC promote a decreased expression of oxidative stress markers (*H O -1* and *H O -2*) and increase expression of antioxidant markers (*SOD1* and *catalase*); increase of E2 and AMH; increase of expression of genes related to development follicles (*Nobox*, *Lhx8*, *Nano3*)*;* increase of number of follicles
Luo et al. (2020)	POF mice by cyclophosphamide	hPMSC		1 × 10^6^/ml hPMSC	tail vein	hPMSCs induce increase of amounts of INHBB and FSHR and reduce the apoptosis of granulosa cells
Yin et al. (2018)	POF mice by ZP3 protein	hPMSC		1 × 10^6^ hPMSC	tail vein	hPMSC increase E2 and AMH levels, while decrease the levels of FSH, LH and AZPAb; increase of expression of *p-Akt;* decrease apoptosis in granulosa cells; decrease of the ratios of Th17/Tc17 and Th17/Treg cells and decrease of the serum levels of IL-17
Ding et al. (2017)	POF mice by cyclophosphamide	hAEC and hAMSC		no dose reported	tail vein	hAMSC promote granulosa cell proliferation better than hAECs
Zhang and Liu (2015)	POF mice by cyclophosphamide and busulfan	hAEC		2 × 10^6^/ml hAEC	tail vein	hAEC inhibit TNF-α-mediated granulosa cell apoptosis; reduced inflammatory reaction in ovaries; promote follicle development; increase cumulus oocyte complexes number; improved ovarian mass and increased the number of follicles; increase the number of pups born
Huang et al. (2020)	Mice with physiological ovarian aging	hAF-MSC			intra-ovary	hAF-MSC increase follicle numbers and improve hormone levels. They increase mRNA and protein expression levels of ovarian markers at four stages of folliculogenesis and inhibit DNA damage genes expression
Lai et al. (2013)	Sterilized mice	hAF-MSC		2–5 × 10^3^ cells	intra-ovary	hAF-MSC restore ovarian morphology. Restored ovaries displayed many follicle-enclosed oocytes at all stages of development. hAFCs survive and differentiate into granulosa cells
Liu et al. (2012)	POF mice by cyclophosphamide	hAF-MSC		1×10^3^ cell spheres/μl	intra-ovary	hAF-MSC have a normal cell cycle distribution and undergo cell division *in vivo*
Xiao et al. (2014)	POF mice by cyclophosphamide and busulfan	AF-MSC		5 × 10^5^ AFC in 5 μL	in ovary	AF-MSC increase the number of primordial and antral follicles and decrease the number of atretic follicles; increase of the number of estrous cycles and the number of litters
Lu et al. (2020)	POI rats by cisplatin	hUC-MSC		2 × 10^6^ hUMSCs	tail vein	hUC-MSC significantly increase the number of normal follicles and greatly reduce the number of atresia follicles. The number of apoptotic theca interstitial cells significantly decrease and the hormone level of E2 increase
Li et al. (2017)	Naturally aging rat	hUC-MSC		1 × 10^6^/ml (1 ml per animal). Second treatment after 48h	tail vein	hUC-MSC increase E2 and AMH while FSH decrease; ovarian structure improved and follicle number increased; ovarian expression of *HGF*, *VEGF*, and *IGF-1* protein elevate significantly
Wang et al. (2020)	POF rat induced by ovarian antigen	hUC-MSC		0.25 × 10^6^/ml, 1.00 × 10^6^/ml, or 4.00 × 10^6^/mL	tail vein	The estrus cycle of rats returne to normal and follicular development is significantly improved after transplantation of UC-MSC. In addition, serum concentrations of 17-estradiol (E2), progesterone (P4), and anti-Mullerian hormone (AMH) increase significantly with treatment. Transplantation of UC-MSC also reduce the apoptosis of granulosa cells. hUC-MSC promote proliferation of granulosa cells in dose dependent manner; upregulate expression of *Bcl-2*, *AMH*, and *FSHR* and downregulated the expression of *caspase-3*
Zheng et al. (2019)	POF mice by cyclophosphamide	hUC-MSC		5 × 10^6^ hUCC	intravenously	hUC-MSC induce increase of E2, AMH, and GnRH levels; prolong estrous; decrease *caspase-3* expression; prevent loss of secondary follicles; increase *TrkA* expression and decrease *FSHR* expression; improve pregnant rate and embryos numbers
Shen et al. (2020)	POI mice by cyclophosphamide	hUC-MSC		1 × 10^6^ cells/mL	tail vein	hUC-MSC induce increase of ovarian weight; increase of E2 and decrease of FSH; increase of number of follicles; mice resume the normal estrous cycle
Zhu et al. (2015)	POF rats by cyclophosphamide	hUC-MSC		1 × 10^6^ cells/ml	tail vein and ovary	hUC-MSC induce increase of the number of secondary and antral follicles; higher level of E2 and lower levels of FSH; fertility is restored, and their offspring develop normally, but the litter size of the tail vein injection group is higher than that of ovary injection group
Yin et al. (2020)	POF mice by cyclophosphamide and busulfan	UC-MSC		1 × 10^6^ UCC	tail vein	UC-MSC increase E2 and AMH level; *HO-1* expressed in UCCs help recover the ovarian function activating the JNK/Bcl-2 signal pathway-regulated autophagy and upregulating the circulating of CD8^+^CD28^−^ T cells
Yang Y et al. (2019)	POF mice by cyclophosphamide	UC-MSC		2 ×10^5^ UCC with or without collagen scaffold	in ovary	Collagen-UC-MSC increase E2 and AMH levels, increase ovarian volume, and number of antral follicles; promote granulosa cell proliferation and ovarian angiogenesis with the increased expression of CD31
Song et al. (2016)	POI rats by cyclophosphamide	hUC-MSC		2 × 10^6^ cells/mL	tail vein	hUC-MSC increase the level of E2 and AMH and decrease the level of FSH; significant increase in secondary follicles; reduction of apoptotic cells
Kim et al. (2018)	Ovariectomized rats	hParietal decidua-MSC and their spheroids		1 × 10^5^ cells	intra-ovary	Parietal decidua-MSC transplantation significantly increase the estradiol level and enhance folliculogenesis-related gene expression levels. Spheroid-cultured PMSCs enhance therapeutic potential via increased engraftment efficiency
Li et al. (2018)	POF mice by cyclophosphamide	hChorionic plate-MSC		2 × 10^6^ cells/kg in 200 µl once week for 4 weeks	tail vein	hChorionic plate-MSC restore the level of E2 and FSH and increase the number of follicles and oocytes
Zhang BF et al. (2018)	POF mice by cyclophosphamide	human placental extracts (HPE)		different doses (0.6-1.2-2.4 ml/kg)	intraperitoneally	HPE induce higher ovarian weight, lower number of atretic follicles, higher serum levels of the hormones E2 and progesterone, and lower apoptosis and serum levels of LH and FSH in granulosa cells
Ding et al. (2020b)	POF mice by cyclophosphamide	hPMSC	CM (no dose reported)	1 × 10^6^ hPMCs	caudal vein	hPMSC promote recovered follicular numbers and increase expression of oocyte markers
Zhang et al. (2017)	POF mice by busulfan	hAEC	hAEC-CM	2 × 10^4^ hAEC and concentrated hAEC-CM (from a total of 2 × 10^4^ cells)	orthotopically/ovary injection	hAEC and hAEC-CM promote healthy and mature follicles in ovaries; increase the expression of *AMH*, *MVH*, *BMP15* and *HAS2*
Yao et al. (2016)	POF mice by busulphan	hAEC	hAEC-CM	4 × 10^6^ hAEC or corresponding CM	IP injection	hAEC or hAEC-CM increase follicle number and expression of both *VEGFR1* and *VEGFR2.* They increase detection of CD34 marker and angiogenesis; induce expression of *SRY* gene; increase the number of litters per pregnancy
Ling et al. (2019)	POF mice by cyclophosphamide	hAMSC	hAMSC-CM	4 × 10^6^ AMSCs in 0.6 ml or 100 µl of hAMSC-CM	hAMSCs in tail vein; hAMSC-CM in ovary	hAMSC decrease *Bax* expression and increase *Bcl-2* and endogenous *VEGF* expression in ovarian cells; they inhibit GC apoptosis, and promote angiogenesis and follicular development
Thabet et al. (2020)	POI rats by cyclophosphamide	AF-MSC	EVs da AF-MSC	0.5 × 10^6^ of AFC in 0.5 ml or the amount of EVs secreted by these cells	in ovary	AF-MSC and EVs equally restore total follicular counts, AMH levels, regular estrous cycles, and fruitful conception, while it both diminish *caspase 3* and *PTEN* levels
Xiao et al. (2016)	POF mice by busulfan	AF-MSC	exosomes from AF-MSC	5 × 10^5^ EGFP-AFC or 125 μg of exosomes proteins (an approximate amount produced by 5 × 10^5^ cells overnight)	in ovary	Exosomes have anti-apoptotic effect on granulosa cells; increase the number of primordial follicles; prevent ovarian follicular atresia; reduce the numbers of atretic follicles. miR-10a directly targets *Bim* and results in the down regulation of *Casp9*, which are crucial factors in apoptotic pathway. miR-146a, miR-17, miR-21a and miR-29b also contribute to anti-apoptosis through targeting various genes involved in apoptotic pathway
Yang et al. (2020)	Old mice	hUC-MSC	exosomes from hUC-MSC	10 mg of hUCC-exos	in ovarian bursa	Exosomes activate oocyte PI3K/mTOR signaling pathway and accelerate follicular development evaluated by related genes; increase oocyte production and improve oocyte quality; increase of number of puppies for female
Hong et al. (2020)	POF mice by cisplatino	hUC-MSC	hUC-MSC-CM	30–50 μl hUCC-CM daily from P5 to P9	intraperitoneally	hUC-MSC-CM decrease apoptosis of oocytes and granulosa cells, and increase *G-CSF*, *GM-CSF*, *Cxcl1*, *Ccl2*, *Ccl7*, and *Il23a* expression
Ding et al. (2020b)	POF mice by cyclophosphamide	hUC-MSC	Exosomes	10^12^ prticles/ml	ovaries	hUC-MSC exosomes induce GC proliferation and decrease ROS. miR-17-5P represses *PARP1*, *γH2AX*, and *XRCC6* by inhibiting *SIRT7*
Yang Z et al. (2019)	POF mice by busulfan	hUC-MSC	MVs from hUC-MSC	150 μg of hUCC-MVs	caudal vein	MVs induce increase of the body weight and number of ovarian follicles; increase of E2 level and decrease of FSH level; upregulate mRNA expression levels of angiogenesis-related cytokines, including *VEGF*, *IGF-1*, and *angiogenin* cytokines (*VEGF*, *IGF*, and *angiogenin*); increase expression of total *AKT*, *p-AKT*, and *VEGF.*

Treated animal models displayed paralleled manifestations to clinic features of human POI patients, mainly oxidative stress and apoptosis of granulosa cells, with the consequence of a significant decrease of the number of follicles, level of hormones, weight of ovaries, and number of offspring, as reported in all papers used in this review.

Physiologically, in the ovary, reactive oxygen species (ROS) are generated during ovulation and hence follicular rupture, which is considered an inflammation-like reaction. Several studies in rat and mouse models with induced POI have reported an increase of ROS levels that reduces oocyte quality due to granulosa cell apoptosis (Seok et al., 2020), which can cause a decrease in the number of follicles and a reduced level of oestrogen (Li et al., 2019) and AMH (Zhang H et al., 2018). Normally, presence of AMH allows the survival of small developing follicles, inhibits excessive follicular stimulation by FSH and acts as a regulator of follicular estrogen production. The AMH allows for the maintenance of an ovarian reserve. Then, fewer ovarian primordial follicles in a POF mouse model may be due to a decreased level of AMH in serum that increases the number of primordial follicles becoming the growing follicles. In this situation, the number of the primordial follicles ultimately decreases resulting in premature depletion of the primordial follicle pool (Zhang H et al., 2018) (Figure 1B), as in human POI or POF.

It is known that the follicles become atretic when 10% of granulosa cells have undergone apoptosis and this suggests that granulosa cell apoptosis plays a vital role in the development of follicular atresia (Zhang H et al., 2018). In fact, in a cyclophosphamide-induced mouse model of POF, there is a decreased expression of the antiapoptotic protein B-cell lymphoma 2 (Bcl-2) and an increased expression of the pro-apoptotic protein bcl-2-like protein 4 (Bax). These effects together induce apoptosis in follicular granulocyte cells and increased follicular atresia (Zhang H et al., 2018). In these animal models, it have been highlighted some metabolic aspects that induce POI or POF. For example, the oxidative stress can involve theca interstitial cells too and induce apoptosis and autophagy. These cells play an important role in folliculogenesis because they provide nutrients and hormones to granulosa cells through the basement membrane *via* vessels (Lu et al., 2020). The result is ovarian failure by a loss of oocyte maturation and granulosa cell luteinization (Seok et al., 2020). Granulosa cell apoptosis in POF mice may also be triggered by the endoplasmic reticulum (ER) stress response. Caspase-12 is the key molecule of regulating ER stress induced apoptosis and it is activated during ER stress. Indeed, studies in these animal models have shown that caspase-12 deficiency mice were resistant to ER stress-induced apoptosis (Li et al., 2019). Furthermore, DNA and proteins can also be damaged by oxidative stress with the consequent disruption of cellular processes (Liu et al., 2012).

In addition to mouse and rat as animal models, the cow could be used as possible and useful model to study the ovarian pathology and potential new treatments. In this species, there are spontaneous reproductive disorders (López-Gatius et al., 2006), mostly due to ovarian abnormalities, among which ovarian failure, that is the most common ovarian disorder (11.45%), followed by adhesions and cysts (6.38 and 5.22%, respectively) (Mekibib et al., 2013). Usually, cows have a dominant follicle that develops to the Graafian follicle stage which subsequently ovulates. After ovulation, this follicle will form the corpus luteum responsible for the secretion of progesterone. If a follicle of at least 8–15 mm is present on two consecutive examinations during the postpartum period and there is absence of cysts or luteal structures, it can be assumed that the cow may be affected by ovarian hypofunction and to have ovarian anovulation (Cremonesi et al., 2020). These animals are healthy but sub-fertile thus causing considerable economic damage to farms.

## Classical Treatment Options

To date, several therapeutic approaches have been applied to POI patients to alleviate complications caused by low oestrogen levels and to improve fertility, among them 1) hormone replacement therapy (HRT), 2) cryopreservation of ovarian tissue and 3) administration of gonadotropins releasing hormone (GnRH) agonists. Hormone replacement therapy is effective for symptoms associated with low oestrogen levels but does not improve fertility (Ding et al., 2020a). Furthermore, this therapy is associated to some negative effects such as thromboembolism, stroke, vaginal bleeding, heart disease, and breast cancer (Kim et al., 2018). For this reason, half of post-menopausal women worldwide live without reproductive hormones, such as oestrogen and progesterone (Kim et al., 2018). Although it has recently been reported that for women under the age of 60 that various risks can be reduced when this therapy is used at an appropriate timing, it still the disadvantage of being ineffective when it is stopped or not used at the correct schedule (Seok et al., 2020). Alternatively, cryopreservation of oocytes and ovarian tissue is a good option for preserving fertility, but the method is an “*ex vivo*” option and does not maintain ovarian function for an extended period (Hong et al., 2020). Finally, the administration of GnRH agonists has been proposed as a potential strategy. Although first-stage results demonstrate a significant reduction in ovarian failure induced by chemotherapy, the protective effects of GnRH agonists remain controversial (Hong et al., 2020).

There are also several other experimental approaches under investigation for POI patients. Among them, the use of phytoestrogens or herbal remedies are reported, but to date no data is available on the mechanism of action and/or long-term safety (Li et al., 2017). Similarly, application of the c-Abl kinase inhibitor imatinib, the sphingosine-based lipid signalling molecule sphingosine-1-phosphate (SIP) and the LH have been reported to prevent premature infertility (Hong et al., 2020), but future confirmatory studies will be needed. Learning from the veterinary field of medicine, cows are experimentally treated with GnRH-based treatments such as the Ovsynch protocol (López-Gatius et al., 2001; Lopez-Gatius et al., 2004; Yaniz et al., 2004) or a progesterone releasing intravaginal device (PRID) (Yániz et al., 2008), but oestrus response in affected cows is very low (usually <30%). Overall, all these methods do not fundamentally improve ovarian function and therefore the development safe and new treatments that can recover normal ovarian function would represent an important advance in reproductive biology.

## Towards Cellular Therapies

The field of regenerative medicine includes tissue engineering and cell therapy. Among the different cells for the treatment of diseases in both human and veterinary medicine, there are mesenchymal stromal cells (MSC) that can be collected from adult and perinatal tissues. Considering a possible transplantation, the source of these cells can be autologous or allogeneic.

Mesenchymal stromal cells are adult non-hematopoietic cells that are present in specific stem cell niches of various organs and tissues. The main properties of MSC are self-renewal and tri-lineage differentiation potential. However, upon *in vivo* administration, true engraftment of transplanted MSC, and as such tissue replacement, has only been documented in a few cases (Duffield et al., 2005; Tögel et al., 2005; Bi et al., 2007; Chimenti et al., 2010). Nevertheless, many studies have demonstrated that transplanted MSC rather act by paracrine/autocrine mechanisms, thereby regenerating injured tissues by means of trophic support. Mesenchymal stromal cell administration can prevent fibrosis and apoptosis, promotes angiogenesis, stimulates the function and differentiation of endogenous stem cells, and even decreases deleterious immune response (Huang et al., 2020). Consequently, the use of MSC has been applied in animal models for various diseases including, for example, myocardial infarction, neurological diseases, and diabetes (Wang et al., 2020). Since MSC also display immunosuppressive and anti-inflammatory properties, they are under investigation for the treatment of various inflammatory diseases, including inflammatory bowel disease, atopic dermatitis, and rheumatoid arthritis (Liao et al., 2019).

Alternative to the direct administration of autologous or allogeneic MSC is the application of MSC-derived factors to the diseased or affected organ or tissue. Although still under debate, it is highly likely that released secretome, that if collected *in vitro* is called conditioned media (MSC-CM) recapitulate the various functions of MSC that harbour their regenerative properties (Hong et al., 2020; Seok et al., 2020). Conditioned medium secreted by MSC is rich in soluble factors and insoluble components represented by extracellular vesicles (EVs) capable to attenuate tissue damage, inhibit apoptosis and fibrosis, promote angiogenesis, and to modulate immune responses (Bruno et al., 2019; Seok et al., 2020). Furthermore, MSC-CM has significant advantages compared to direct MSC application, including that 1) it can be lyophilized and transported more easily, 2) it reduces rejection problems between donor and recipient, and 3) it eliminates potential tumorigenic effects such as uncontrolled differentiation, increased metastatic capacity of tumour cells and epithelial-mesenchymal transition of tumour cells (Hong et al., 2020). The soluble factors in MSC-CM encompass chemokines, cytokines, and growth factors. On the other end, MSC-derived EVs are membranous structures with lipid bilayers of nanometre size that are normally released into the extracellular space (Thabet et al., 2020). Extracellular vesicles can be subcategorized in exosomes, which are the major subclass of EVs with sizes ranging from 50 to 120 nm and shedding vesicles or microvesicles that represent larger-sized vesicles (50–1,000 nm) (Thabet et al., 2020). Extracellular vesicles are rich in proteins, lipids, RNAs and microRNAs (miRNAs). MicroRNAs regulate diverse cellular processes by suppressing the expression of genes mainly via inhibition of their messenger RNAs (mRNAs) (Thabet et al., 2020). In this way, numerous studies have already demonstrated that EVs have intrinsic therapeutic effects in various diseases such as wound healing, inflammation, hypertension, cardiovascular disease, brain injuries and tumours (Yang et al., 2020). Similar to CM, EVs are very promising as therapeutic agents as compared to MSC, especially with regard to their low immunogenicity, lack of tumorigenicity, high clinical safety and low ethical risk compared to the cells of origin (Yang et al., 2020).

In addition to a general preference of using secretomes instead of live cell preparations, regenerative medicine is also exploring the use of alternative cellular sources in comparison to those considered to date as the gold standard, such as bone marrow and adipose tissue. Human bone marrow-MSC were the first cell type that was shown to improve ovarian function and structure in a rat model with ovarian hypo functionality caused by chemotherapy. Subsequently, other sources such as adipose tissue (Kim et al., 2018), human menstrual blood, endometrium (Li et al., 2018) and skin (Seok et al., 2020) have been applied to rat models of ovarian dysfunction, with similar successes. Furthermore, MSC derived from bone marrow and adipose tissue also displayed positive effects on damaged ovarian tissue in a POF mouse model, thereby promoting an increase in E2 level, suppressing apoptosis and promoting angiogenesis (Kim et al., 2018).

From a clinical point of view, the collection of bone marrow and adipose tissue requires invasive procedures. Moreover, the actual cell yield for transplantation is rather low to insufficient due to decreased proliferation and a reduced differentiation capacity with age for these adult somatic cell types (Lange-Consiglio et al., 2012). An alternative source could be pluripotent stem cells, including embryonic stem cells (ESC) and induced pluripotent stem cells (iPSC), which proliferate rapidly and can give rise to three germ layer lineages. Another interesting feature of pluripotent stem cells is their ability to differentiate into germ cells, which have undergone meiosis and produce gametes (Lai et al., 2013). However, murine ESC *in vitro* have not supported the completion of meiosis and ESC-derived oocyte maturation ultimately fails *in vitro* (Lai et al., 2013). Furthermore, these cells do have limited use in clinical trials due to ethical concerns, allograft rejection problem, and risk of teratoma formation (Lin et al., 2015). Recently, the use of perinatal derivatives (PnD) in the development of cellular and acellular therapies for ovarian diseases has become a topic of interest in medical research.

## Perinatal Derivatives (PnD)

The term “perinatal” refers to the tissues associated with birth that are collected from the term placenta (chorionic villi, chorionic plate) and extra-foetal tissues (including amniotic and chorionic membrane, amniotic fluid, and umbilical cord). The term derivatives refers to both living cells as well as their secretome or conditioned medium, including extracellular vesicles (Silini et al., 2020). Perinatal tissues, as compared to BM, represent an important source of MSC because they are a readily available source whose collection is painless and non-invasive, with minimal ethical issues as they are usually discarded at birth (Papait et al., 2020). In general, perinatal MSC show similar characteristics of bone marrow-MSC, including proliferation potential, self-renewal capacity, differentiation potential into multiple cell types, and chemotactic migration potential to sites of injury (Ling et al., 2019). Interestingly for regenerative medicine purposes, their higher proliferation rate as compared to adult tissue derived MSC can be explained by a more primitive developmental stage for these cells (Ilancheran et al., 2009). In addition, long-term *in vitro* culture does not seem to alter their phenotype and genetic stability (Sabapathy et al., 2014), which is not always the case for bone marrow-MSC.

In addition to the same characteristics of MSC, PnD also display several phenotypic characteristics of ESCs (Ling et al., 2019). For example, amniotic fluid-MSC express octamer-binding transcription factor 4 **(**OCT4), but unlike ESC they do not develop into teratomas (Xiao et al., 2014). It has also been suggested that several types of PnD can be used for allogeneic transplantation without rejection because they show hypo immunogenicity and the ability to modulate immune responses (Li et al., 2017; Ling et al., 2019). Compared to bone marrow-MSC, that are not able to exert suppressive effects if they are not previously exposed to inflammatory stimuli, PnD do not require “licensing” with inflammatory stimuli such as interferon (IFN) and tumor necrosis factor-α (TNF-α) (Lange-Consiglio et al., 2020; Papait et al., 2020). Moreover, PnD produce higher levels of cytokines and growth factors as compared to bone marrow-MSC and adipose-MSC, including granulocyte colony-stimulating factor (G-CSF), regulated on activation, normal T cell expressed and secreted (RANTES) and interleukin (IL)-6/-8/-10 (Seok et al., 2020). Therapeutic effects of PnD have already been highlighted in hepatic diseases, optic nerve crush injury (Seok et al., 2020), tendon lesion (Lange-Consiglio et al., 2013) and in many other diseases (Silini et al., 2017). These therapeutic effects include anti-fibrosis, anti-inflammation and anti-apoptosis effects (Lange-Consiglio et al., 2016; Kim et al., 2019). Given this knowledge, exploring the use of PnD as a potential innovative therapy in ovarian diseases is highly encouraged.

## Materials and Methods

A search was performed on PubMed to identify all papers describing the use of PnD by means of the following combination of terms, within the 2004–2021 time limit: (placenta OR placental OR “perinatal tissue” OR “neonatal tissue” OR decidua OR amnion OR “amniotic fluid” OR “amniotic membrane-derived” OR “human amniotic membrane” OR “umbilical cord” OR “Wharton’s jelly” OR “Wharton jelly” OR “Whartons jelly” OR chorion OR “chorionic membrane” OR “fetal membrane” OR “fetal tissue” OR “villous stroma”) AND (“stem cells” OR “progenitor cells” OR “stroma cells” OR “stromal cells” OR “mesenchymal cells” OR “amnion epithelial cells” OR “amniotic epithelial cells” OR “amniotic membrane-derived cells” OR “amniotic membrane transplantation” OR “extracellular vesicles” OR exosomes OR microvesicles OR secretome OR “conditioned medium” OR scaffold OR “protein extracts” OR “extract”) NOT (“umbilical cord blood” OR “cord blood” OR hematopoietic OR haematopoietic OR review [Publication Type]) AND (animal OR “*in vivo*” OR preclinical OR pre-clinical OR mouse OR mice OR rat OR rodent OR rabbit OR sheep OR ovine OR swine OR pig OR horse OR equine OR cow OR bovine OR dog OR canine OR fish OR primate OR primates OR organoids OR “decellularized matrix” OR “de-cellularized matrix” OR “decellularised matrix” OR “de-cellularised matrix”). Articles in languages other than English, guidelines, reviews, and scientific video protocols were excluded.

Fifty-six manuscripts focusing on reproductive diseases were selected covering ovary (*n* = 39), utero (*n* = 9), testicle (*n* = 6) and mammary (*n* = 2) diseases. Considering the limited number of manuscripts using PnD in different reproductive disorders, in this review, the attention was focused on the therapies with PnD in the treatment of ovarian diseases.

## Ovarian Diseases and PnD Therapy

Several recent discoveries have shown the potential of cell therapy to restore chemotherapy-induced ovarian failure in mouse or rat animal models. Chemotherapy can induce granulosa cell apoptosis, follicular loss, vascular damage and tissue fibrosis, ultimately leading to ovarian failure (Ling et al., 2019). Over the years, cellular therapy has been suggested to display positive therapeutic effects via different mechanisms. From direct differentiation into oocytes or granulosa cells when aiming at ESC/iPSC therapy, to restoration of ovarian function through the paracrine effect when aiming at MSC therapy (Ling et al., 2019), with the latter obviously being most accessible for clinical application. As described above, considering the limitations using adult and embryonic stem cells, several studies have recently investigated the use of human perinatal cells derived from amniotic fluid, amniotic membrane, umbilical cord and placenta, including chorionic plate and villous chorion. All these PnD share the following characteristics: they are easy to obtain, there is abundant starting material, they display low immunogenicity, they are easy culture, they do not display oncogenicity and there are no ethical restrictions (Liu et al., 2012). Characteristics of currently used perinatal cells derived from different sources and their putative mode of action in the therapeutic treatment of POI or POF are thereafter described and summarized in Table 1.

Placenta MSC (PMSC). Transplantation of PMSC can significantly restore ovarian function by altering 1) the expression levels of folliculogenesis-related genes, 2) the number of follicles (Kim et al., 2018) and 3) by promoting ovulation with more oocytes collected after 6 weeks of PMSC transplantation versus control (Li et al., 2018). These pre-clinical observations in animal models are related to the anti-apoptotic (Zhang H et al., 2018) and antioxidant effects of PMSC as well as their homing activity, which positively impacts POI by the secretion of epidermal growth factor (EGF). The EGF secretion will inhibit ROS by upregulating nuclear factor erythroid 2–related factor 2 (NRF2) and heme oxigenase-1 (HO-1) expression, and by inhibiting phosphatase and tensin homolog **(**PTEN) expression (Ding et al., 2020b). There is also evidence that PMSC transplantation decreases apoptosis of granulosa cells by increasing the inhibin B (INHBB) levels, as it was shown that low levels of INHBB increase apoptosis of granulosa cells (Luo et al., 2020). Furthermore, PMSC transplantation also attenuates granulosa cell apoptosis by inhibiting excessive activation of the ER stress pathway. In this context, it has been shown that caspase-12 is significantly inhibited in the ovaries of POF mice upon PMSC transplantation (Li et al., 2019). Also the phosphatidylinositol 3-kinase/protein-kinase B (PI3K/Akt) signalling pathway plays an important role in folliculogenesis and controls the survival, loss, and activation of primordial follicles in the oocyte (Yin et al., 2018). After PMSC transplantation in POF mice, the PI3K/Akt signalling pathway promotes the recovery of ovarian function by modifying the ratios of Th17/Tc17 and Th17/Treg cells, which are involved in the pathogenesis of inflammatory and autoimmune diseases (Yin et al., 2018). In summary, PMSC could facilitate the development of follicles and oocytes in ovarian disease by multiple mechanisms, including the reduction of cellular stress, the prevention of apoptosis and the modulation of inflammatory responses (Li et al., 2018). Further evidence comes from the interesting observation that PMSC cultured in 3D spheroidal cultures, as compared to 2D cultured PMSC, significantly increase the number of ovarian follicles, the production of E2 and the expression levels of folliculogenesis-related genes in a shorter time frame, when grafted in mice with chemotherapy-induced POF (Kim et al., 2018). Moving towards the application of acellular PnD therapeutic products, administration of human placental extracts (hPE) have been shown to favour weight gain of the ovary, a reduction of the number of atretic follicles, an increase of serum levels of the hormones E2 and progesterone, a reduction of serum levels of FSH and a decrease of apoptosis in granulosa cells. Furthermore, it has also been shown that treatment with hPE downregulates phospho-Rictor (*p*-Rictor), BCL2 associated agonist of cell death (Bad), Bax, peroxisome proliferator activated receptors (PPAR) and upregulates *p*-Akt and phosphor-forkhead boxO3a (*p*-Foxo3a). These effects together can promote protection of granulosa cells from apoptosis, prevent follicular atresia, and relieve symptoms of cycloheximide induced ovarian damage (Zhang BF et al., 2018).

Amniotic membrane cells (AMC). For AMC it is important to distinguish between amniotic epithelial cells (AEC) and amniotic mesenchymal cells (AMSC). Administration of both cell types has demonstrated a positive effect on POF. Interestingly, AMSC displayed a greater therapeutic activity than AEC, especially in the cycloheximide mouse model. This is most likely due to the large amount of collagen secreted by AMSC, which promoted enhanced engraftment and increased proliferation of transplanted AMSC (Ding et al., 2017). The latter is further supported *in vitro*, where AMSC favour a better proliferation rate of granulosa cells (Ding et al., 2017). Notwithstanding, AEC transplantation was also able to inhibit TNF-α-mediated apoptosis in granulosa cells and to reduce inflammation in chemotherapy-induced ovarian damage (Zhang and Liu, 2015).

Amniotic fluid MSC (AF-MSC). Cheng et al. (2012) reported that hAF-MSC cultured in medium containing 5% porcine follicular fluid can differentiate into oocyte-like cells. In addition, these cells were able to restore folliculogenesis that included four stages: primordial germ cells and oogonia formation, follicle formation, follicular growth and follicular maturation. In each of these stages, AF-MSC displayed increased ovarian marker expression: primordial germal cell oogonia (Forkhead Box L2: FOXL2 and cytochrome P450 family 19 subfamily A member: CYP19A1), follicle formation (MutS Homolog 4: MSH4 and stromal antigen 3: STAG3), follicular growth (growth differentiation factor 9: GDF9 and AMH) and follicular maturation (follicle stimulating hormone receptor: FSHR and bone morphogenetic protein 15: BMP15). Moreover, these oocyte-like cells can increase the level of E2 and AMH (Huang et al., 2020). These hormones strongly correlate with the size of the follicular pool (Lai et al., 2013) and repress apoptosis in granulosa cells in animals with ovarian physiological aging (Huang et al., 2020). Although further research is needed, AF-MSC may sustain the number of healthy ovarian follicles in POF mice either by promoting *de novo* folliculogenesis, by inhibiting follicular atresia or a combination of both (Xiao et al., 2014).

Umbilical cord-MSC (UC-MSC). Transplantation of UC-MSC in a POF rat model induced the return to normal oestrus cycle with follicular development, increased serum concentrations of E2, progesterone (P4) and AMH, reduced apoptosis and increased proliferation of granulosa cells. Furthermore, transplantation of UC-MSC up-regulated increased expression of Bcl-2, AMH and FSHR in the ovary of POF rats and downregulated the expression of caspase-3 (Zheng et al., 2019; Wang et al., 2020). Umbilical cord cells can also alleviate POI injury by inhibiting apoptosis of theca-interstitial cells by suppression of the autophagy signalling pathway adenosine monophosphate-activated protein kinase/mechanistic target of rapamycin (AMPK/mTOR) (Lu et al., 2020) and increasing nerve growth factor (NGF) and nerve growth factor receptor (TrkA) levels. The TrkA receptor predominantly activates PI3K and mitogen-activated protein kinase (MAPK) to promote cell survival and proliferation (Zheng et al., 2019). The PI3K/Akt signalling pathway regulates oocyte growth and the survival and development of primordial follicles, promotes the proliferation and differentiation of granulosa cells, and inhibits apoptosis, and these processes are critical for the normal development and physiological functions of the ovaries (Zheng et al., 2019). Furthermore, UC-MSC can reduce the degree of apoptosis and improve the endocrine function of mouse ovaries (Shen et al., 2020) restoring fertility and offspring that develop normally (Zhu et al., 2015). Finally, the mechanism of action of UC-MSC may also be attributable to the action of the HO-1 gene expressed by these cells. The HO-1 has potent anti-inflammatory, antioxidant, and immunoregulatory properties. Moreover, HO-1 also participates in the physiology of the ovary itself, as well as in the secretion of gonadotropins from the pituitary gland. Studies have demonstrated that HO-1 helps recover the ovarian function of POF mice with UC-MSC transplantation *via* activation of the Jun N-terminal kinase (JNK)/Bcl-2 signal pathway. This pathway regulates autophagy and increases the number of circulating CD8^+^CD28^−^T cells. These CD8^+^CD28−T cells display typical immunosuppressive function and can induce immunological tolerance in transplantation, demonstrating immunologic suppression in organ transplants (Yin et al., 2020). Interestingly for future research, UCC have recently been grafted in ovary of POF induced mice in with the support of a collagen scaffold, hereby demonstrating a greater therapeutic effect compared the UC-MSC grafting without scaffold support (Yang Y et al., 2019).

## The Importance of PnD Secretomes in Ovarian Diseseas

**FIGURE 2:**
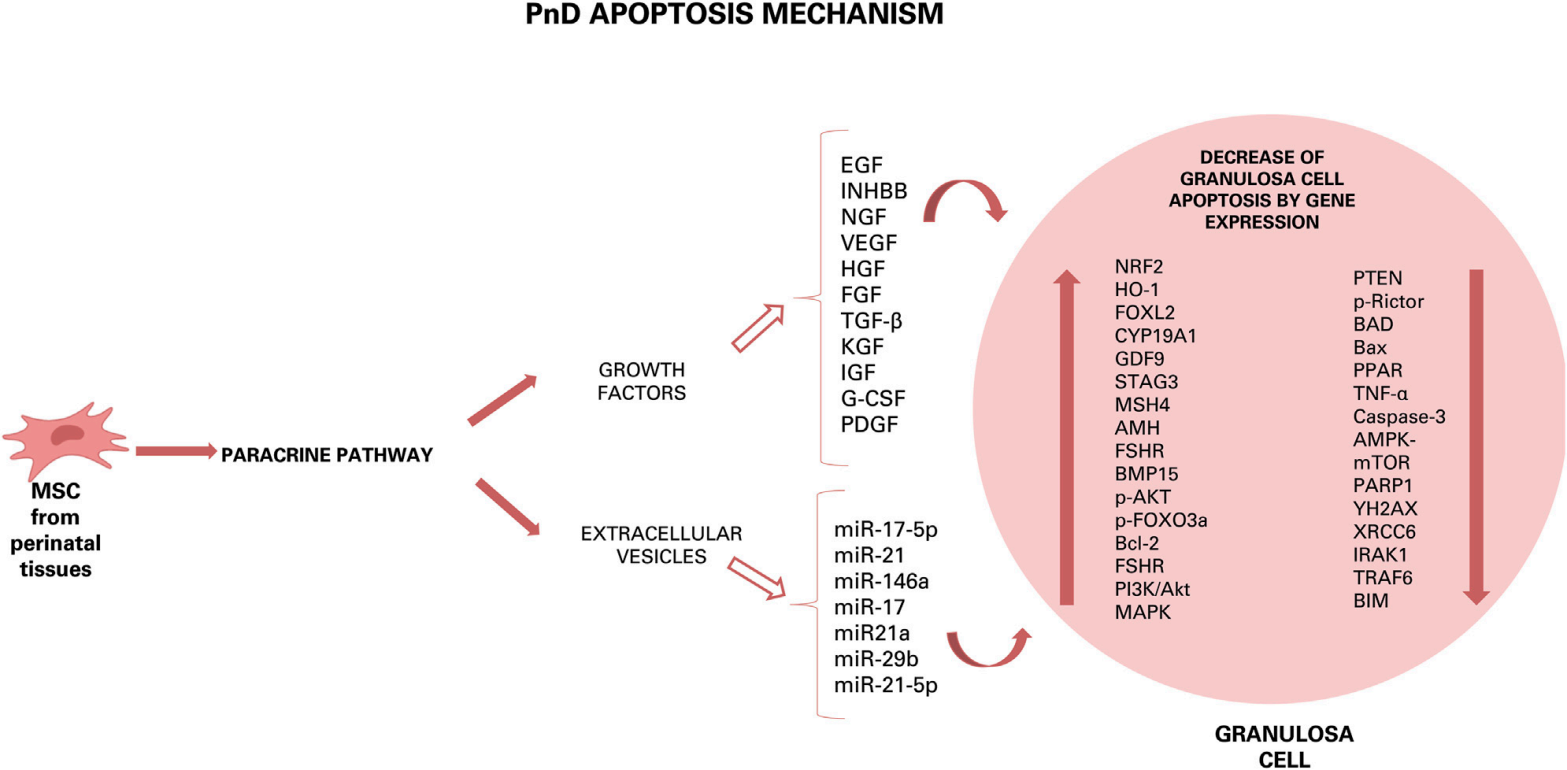
Hypothesis of action of PnD and their effect on ovarian diseases. Growth factors and components of EVs can induce different effects: anti-apoptotic, antioxidant, pro-angiogenetic, restoration of hormonal levels, autophagy and anti-inflammatory by down- or up-regulation of some gene expression or by increase or decrease of some factor. The results of this effects are the increase of cumulus oocyte complexes number, of ovarian mass, of number of follicles, of number of estrous cycles and, then, of number of pups born.

Although recent studies have shown that AMSC transplantation in rats with chemotherapy-induced POI restored ovarian function, grafted cells were only detectable in the interstitium of the ovaries and not in the follicles. Indeed, these cells did not express the typical oocyte and granulosa cell markers, which are zona pellucida sperm-binding protein 3 (ZP3) and FSHR, respectively. These results suggest that grafted AMSC do not differentiate into oocytes or granulosa cells but are able to improve ovarian function *via* a paracrine mechanism (Ling et al., 2019). This paracrine effect is most likely related to the secretion of growth factors able to reduce apoptosis of granulosa cells and to recover follicular development with increase of serum levels of AMH and E2 and decreased FSH (Song et al., 2016) (Figure 2). Several studies have shown that also the AM is a source of multiple growth factors, including vascular endothelial growth factor (VEGF), hepatocyte growth factor (HGF), fibroblast growth factor 2 (FGF2), transforming growth factor beta (TGF-ß), keratinocyte growth factor (KGF), insulin growth factor (IGF-1) and G-CSF, which were highly likely to play important roles in repairing ovarian injury and restoring ovarian function in animals with POI (Zhang et al., 2017; Li et al., 2018). These growth factors have been shown to inhibit apoptosis and to stimulate proliferation of granulosa cells, to upregulate Bcl-2 expression, to downregulate Bax expression, and to promote local VEGF expression in the ovaries of POI rats with the result of angiogenesis and follicular growth (Ling et al., 2019). Next to the AM, also AF-MSC secrete molecules among which TGF-b (Yoon et al., 2010), vascular endothelial growth factor and glia cell-derived neurotrophic factor (GDNF), which are required for follicular development and can inhibit follicular cell apoptosis and follicle atresia (Xiao et al., 2014).

Further evidence for the importance of the secretome of grafted cell types comes from the observation that transplantation of UCC improve the ovarian reserve function of perimenopausal rats through secretion of cytokines such as VEGF, HGF and IGF-1, whose expression distinctly increased after umbilical cord-MSC transplantation. VEGF is a powerful survival factor for ovarian granulosa cell apoptosis and ovarian follicular atresia (Li et al., 2017), while HGF, expressed both in thecal cells and granulosa cells of rat ovaries, may play its function as a modulator of the mesenchymal-epithelial cell transition between theca cells and granulosa by facilitating cell proliferation and steroid hormone production. A complete HGF system also supports granulosa cells growing via an anti-apoptotic effect. The IGF-1 is expressed in growing granulosa cells and healthy follicle and is necessary for the proliferation of granulosa cells at the early stage of folliculogenesis (Li et al., 2017).

**FIGURE 3:**
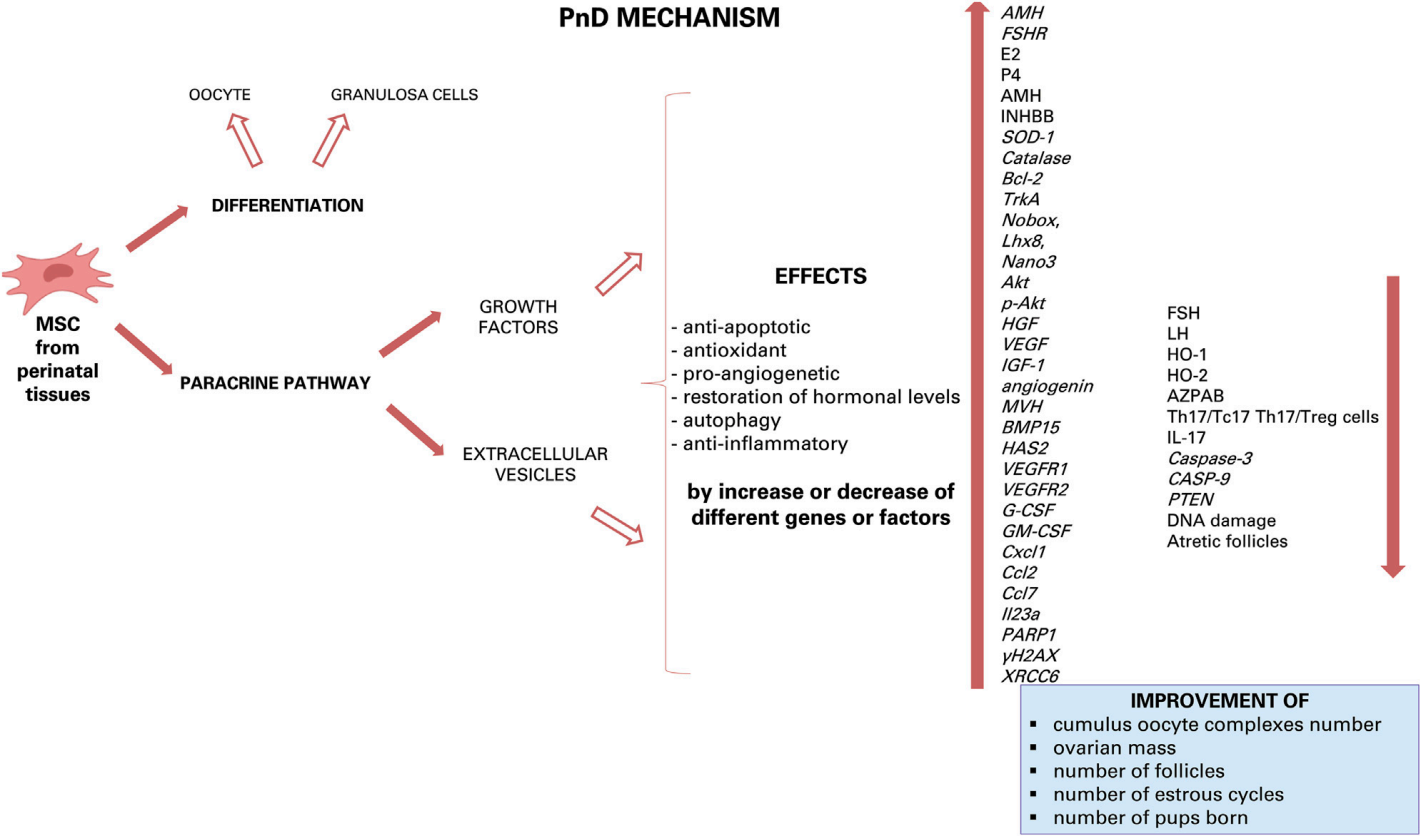
Hypothesis of apoptosis mechanism of PnD. Inside the granulosa cells, some growth factors and some miRNAs can induce down- or up-regulation of some gene expression.

Concluding, to data there are many substances produced by various PnD that can have a beneficial effect by paracrine action on ovarian disease, with each of them triggering important pathways in cell/tissue protection and/or regeneration. Some effects of these substance can be anti-apoptotic, antioxidant, pro-angiogenetic, restoring of hormonal levels, autophagic and-anti-inflammatory (Figure 3). The future goal will be to further dissect each of these modes of action and to derive an optimal therapeutic scheme.

### Ovarian Diseases and Conditioned Medium Therapy

In the context that PnD secrete many molecules and act by paracrine mechanism, as discussed above, the application of CM derived from perinatal (stem) cells has gained interest as a novel therapy for POI or POF. Several studies have indeed shown the potential use of CM over cell transplantation. For example, injection of CM secreted by AEC significantly increased the number of secondary and mature follicles, as well as upregulated follicle growth related genes expression (AMH; mouse vasa homologue: MVH; BMP15 and hyaluronan synthase 2: HAS2) (Zhang et al., 2017). Furthermore, daily (for 5 days) intraperitoneal administration of CM derived from UC-MSC daily upregulated G-CSF expression in granulosa cells. This factor plays important roles in ovulation, oocyte maturation, development of preimplantation embryos, trophoblast invasion and attenuates oxidative stress-induced cell apoptosis through the PI3K/Akt pathway (Hong et al., 2020). According to Akdemir et al. (2014), G-CSF can also reduce follicle loss. Lastly, administration of CM derived from AEC improved follicle number and fertility in mice with POI. This beneficial effect was induced by regulating expression of VEGFA and its receptors, thus inducing angiogenesis and increased follicular growth (Yao et al., 2016). Since it has been demonstrated that AEC secrete a variety of growth factors, such as epidermal and fibroblast growth factors (HB-EGF, EGF- 2, bFGF, FGF-4, FGF-6, and FGF-7), angiogenic growth factors (VEGF, VEGF-D, VEGF-R2, and VEGF-R3), insulin like growth factors (IGF-1, IGF-ISR, IGFBP-1, and IGFBP-4) and platelet-derived growth factors (PDGF-AA, PDGF-BB, PDGFRa, and PDGFRb), ovaries damaged by chemotherapy would need not only VEGFA for restoration, but most likely also other growth factors secreted by AEC (Yao et al., 2016).

### Ovarian Diseases and Extracellular Vesicle Therapy

With the recognition that conditioned medium, and thus secreted factors, are an important key mechanism of the therapeutic action of cellular therapies, also the paracrine activity of EVs has gained increasing interests (Liu et al., 2012). For example, transplantation of EVs secreted by UCC into the ovary of mice with chemotherapy-induced POI promotes angiogenesis and formation of new blood vessels. This was correlated with increased mRNA expression levels of VEGF, IGF-1 and angiogenin in the ovaries of POI-EV mice as compared to non-treated POI mice (Yang Z et al., 2019).

Among the different types of EVs, exosomes are powerful cell-to-cell communicators with low immunogenicity and no tumorigenicity. These small 40–150 nm EV have a cargo consisting of several molecules including mediators of inflammation, tropic factors, signalling molecules, mRNA, miRNA and long non-coding RNA (lncRNA) (Yang et al., 2020).

Among this large cargo of different types of factors, it has been shown that exosomes released by human umbilical MSC suppress apoptosis of ovarian granulosa cells and regulate the immune response by miRNAs (Sun et al., 2017; Ding et al., 2020b). One miRNA that is highly expressed in exosomes derived from hU-MSC and involved in the therapeutic effect on ovary is miR-17-5P. Injection of this miRNA increases follicle number, ovarian size and foetus number (Ding et al., 2020b). In addition, miR-17-5P restores ovarian function, reduces oxidative stress in granulosa cells by inhibiting the expression of mRNA SIRT7 and its downstream target genes [poly (ADP-ribose) polymerase 1: PARP1; γH2A histone family member X: γH2AX and X-Ray Repair Cross Complementing 6: XRCC6]. These genes are critical mediators of DNA repair: PARP1 is involved in repairing DNA damage, follicular development, and atresia formation (Ding et al., 2020b). In addition, PARP inhibition reduces ROS production; γH2AX is involved in embryonic development, stem cell self-renewal and aging; XRCC6 protects cells from DNA damage and represses BAX induced apoptosis (Ding et al., 2020b).

Another miRNA, which is abundant in AF-MSC, is miRNA-21 and is directly involved in ovarian physiology (Thabet et al., 2020). Treatment with AF-MSC and EVs can be superimposed as in both cases there is an improvement in follicular regeneration thanks to the restoration of AMH levels, an increase in follicles, regular oestrus cycles and conception in rat models with premature ovarian dysfunction. This action occurs by decreasing the expression of PTEN and PI3K/PTEN and caspase 3 proteins (Thabet et al., 2020). Others interesting miRNAs abundantly present in exosome secreted from AF-MSC are miR-146a and miR-10a, which inhibit apoptosis in damaged granulosa cells and prevent follicle atresia in mice with POF due to chemotherapy (Xiao et al., 2016). It has been shown that miR-146a regulates apoptosis *via* the downregulation of the expression of target genes interleukin-1 receptor-associated kinase 1 (Irak1) and TNF receptor associated factor 6 (Traf6), while miR-10a modulates cell apoptosis through the suppression of pro-apoptotic factor, Bcl-2-like protein 11 (Bim) (Xiao et al., 2016). Other miRNAs, such as miR-17, miR-21a and miR-29b have anti-apoptotic action by downregulating the expression of some genes and thus preventing follicular atresia (Xiao et al., 2016). Additional evidence from *in vitro* studies have shown that the increased recruitment of primordial follicles was related to miR-146a-5p or miR-21-5p that stimulate the oocyte PI3K and mTOR signalling (Yang et al., 2020). Concluding, all these studies evidence the efficacy of MSC-derived exosomes in preclinical animal models of reproductive diseases, and, although more research is needed, may provide effective treatments for reproductive diseases.

## Conclusion and Limits

Reducing infertility rates in humans and animals requires ongoing research efforts. This review highlights how research is focusing on stem cell therapy, mainly with PnD, which exert protective effects primarily through the paracrine signalling. The results of the papers used for this review suggest that PnD reduce oxidative stress and apoptosis of the granulosa cells in injured ovarian tissue, promoting the recovery of the oestrus cycle and improvement of endocrine function in treated animals. The findings may provide a theoretical basis for infertile patients who may benefit from PnD treatment in the future.

However, to date, the potential of cell therapy to restore ovarian failure has been tested in mouse or rat animal models that do not fully offer realistic disease models as they do not share the heterogeneity of the human population including genetics and physiological variations, as well as the complex interactions of these with the environment. Therefore, many effects remain to be explored before the possible translation of PnD therapies in human medicine. It would be necessary to employ large animal models to answer several key questions, including the optimal route of administration as it is not yet clear which path(s) allows optimal engraftment of injected cells. Usually, in the rat or mouse model, the route of administration is the tail vein that is not overlapping in human species. A hypothetical large animal could be the pig that presents the advantage of having similarities with the human in terms of gastrointestinal anatomy, metabolism and physiology. For this reason, pigs are widely used as models in organ transplantation and other surgical procedures or as preclinical models in drug discovery and numerous naturally occurring and generated genetic models of human disease (Ribitsch et al., 2020). Another large animal could be the cow that is affected by spontaneous ovarian pathology but,unfortunately, it has never been treated with MSC from any source.

Moreover, it must also be determined whether a single dose is sufficient or whether multiple doses need to be administered. However, in case of the latter, it is not clear at the moment whether risks of adverse immune reactions may arise, especially in human. Regarding the PnD, it would be important to know if different time window for the collection and different culture system of the cells of origin can influence the efficacy of these cells. In addition, considering the paracrine mechanism of action of MSCs, the use of CM or EVs, which can reduce some risks due to cell administration of allogeneic cells, must be further explored.

The mode of isolation and storage of the EVs, the dosage, the route and frequency of their administration, and the safety of post-transplantation have yet to be studied. In addition, it would be important to determine the appropriate modality of delivery of their miRNAs and the timing for treatment. According to data by Xiao et al. (Xiao et al., 2016), the protection of granulosa cells against apoptosis is crucial within 72 h after chemotherapy treatment to prevent POF. It may even be beneficial to administer miRNAs prior to chemotherapy treatment to achieve maximum efficacy.

Furthermore, there are still some obstacles to the application of exosomes for the treatment of POI or for other pathologies: it is necessary to identify a less expensive method of production and purification and to better study the immunomodulatory and homing effects (Ling et al., 2019; Ding et al., 2020a).

Moreover, in future therapies, the use of miRNAs alone is envisaged, but miRNAs play complicated roles in maintaining homeostasis, and their systematic administration in high doses could cause serious side effects (Xiao et al., 2016). Indeed, EVs induce angiogenesis and, as it is well known, angiogenesis plays a critical role in tumour growth, and it is uncertain whether high doses of EVs can cause serious adverse effects, such as ovarian cancer. Therefore, more work on the efficiency and safety of the *in vivo* use of EVs must be done before it can be applied in clinical practice (Yao et al., 2016).

## Glossary

AEC: amniotic epithelial cells
AF: amniotic fluid
AF-MSC: amniotic fluid mesenchymal stromal cells
AM: amniotic membrane
AM-MSC: amniotic membrane-MSC
AMH: anti-Mullerian hormone
AMPK/mTOR: adenosine monophosphate-activated protein kinase/mechanistic target of rapamycin
AMSC: amniotic mesenchymal cells
AT: adipose tissue
Bad: BCL2 associated agonist of cell death
Bax: bcl-2-like protein 4
Bim: Bcl-2-like protein 11
BM: bone marrow
BMP15: bone morphogenetic protein 15
CM: conditioned media
CYP19A1: cytochrome P450 family 19 subfamily A memberE2: 17β-estradiol
EGF: epidermal growth factor
ER: endoplasmic reticulum
ESC: embryonic stem cells
EV: extracellular vesicles
FGF2: fibroblast growth factor 2
FOXL2: Forkhead Box L2
FSH: follicle-stimulating hormone
FSHR: follicle stimulating hormone receptor
G-CSF: granulocyte colony-stimulating factor
GDF9: growth differentiation factor 9
GDNF: glia cell-derived neurotrophic factor
GnRH: gonadotropins releasing hormone
HAS2: hyaluronan synthase 2
CP: human chorionic plate
hCV: human villous chorion
HGF: hepatocyte growth factor
HO-1: heme oxigenase-1
hPE: human placental extracts
IFN: interferon
IGF-1: insulin growth factor
IL: interlukin
INHBB: inhibin B
iPS: induced pluripotent stem cells
Irak1: interleukin-1 receptor-associated kinase 1
JNK: Jun N-terminal kinase
KGF: keratinocyte growth factor
LH: luteinizing hormone
MAPK: mitogen-activated protein kinase
miRNAs: microRNAs
mRNAs: messenger RNAs
MSC: mesenchymal stromal cells
MSH4: MutS Homolog 4
MVH: mouse vasa homologue
NGF: nerve growth factor
NRF2: nuclear factor erythroid 2–related factor 2
OCT4: octamer-binding transcription factor 4 physiological ovarian aging
PARP1: poly [ADP-ribose] polymerase 1
PDGF: platelet-derived growth factors
p-Foxo3a: p-Akt and phosphor-forkhead boxO3a
PI3K/Akt: phosphatidylinositol 3-kinase/protein-kinasi B
PMSC: Placenta MSC
PnD: perinatal derivatives
POF: premature ovarian failure
POI: premature ovarian insufficiency
PPAR: peroxisome proliferator activated receptors
p-Rictor: phospho-Rictor
PRID: progesterone releasing intravaginal device
PTEN: phosphatase and tensin homolog
RANTES: regulated on activation, T cell expressed and secreted
ROS: reactive oxygen species
SIP: sphingosine-1-phosphate
STAG3: stromal antigen 3
TGF-ß: transforming growth factor-ß
TNF-α: tumor necrosis facor-α
Traf6: TNF receptor associated factor 6
TrkA: nerve growth factor receptor
UC: umbilical cord
UC-MSC: umbilical cord-MSC
VEGF: vascular endothelial growth factor
XRCC6: X-Ray Repair Cross Complementing 6
ZP3: zona pellucida sperm-binding protein 3
γH2AX: γH2A histone family member X
